# The hippocampus as the switchboard between perception and memory

**DOI:** 10.1073/pnas.2114171118

**Published:** 2021-12-08

**Authors:** Matthias S. Treder, Ian Charest, Sebastian Michelmann, María Carmen Martín-Buro, Frédéric Roux, Fernando Carceller-Benito, Arturo Ugalde-Canitrot, David T. Rollings, Vijay Sawlani, Ramesh Chelvarajah, Maria Wimber, Simon Hanslmayr, Bernhard P. Staresina

**Affiliations:** ^a^School of Computer Science and Informatics, Cardiff University, Cardiff CF24 3AA, United Kingdom;; ^b^School of Psychology and Centre for Human Brain Health, University of Birmingham, Birmingham B15 2TT, United Kingdom;; ^c^cerebrUM, Département de Psychologie, Université de Montréal, Montreal, QC H2V 259, Canada;; ^d^Princeton Neuroscience Institute, Princeton University, Princeton, NJ 08544;; ^e^Department of Psychology, Princeton University, Princeton, NJ 08540;; ^f^Laboratory of Cognitive and Computational Neuroscience, Center for Biomedical Technology 28223 Madrid, Spain;; ^g^Faculty of Health Sciences, King Juan Carlos University 28933 Madrid, Spain;; ^h^Neurosurgery Service, Hospital Universitario La Paz 28046 Madrid, Spain;; ^i^Epilepsy Monitoring Unit, Neurology and Clinical Neurophysiology Service, Hospital Universitario La Paz 28046 Madrid, Spain;; ^j^School of Medicine, Universidad Francisco de Vitoria 28223 Madrid, Spain;; ^k^Complex Epilepsy and Surgery Service, Neurophysiology Department, Queen Elizabeth Hospital, Birmingham B15 2GW, United Kingdom;; ^l^Complex Epilepsy and Surgery Service, Neuroradiology Department, Queen Elizabeth Hospital, Birmingham B15 2GW, United Kingdom;; ^m^Complex Epilepsy and Surgery Service, Neurosurgery Department, Queen Elizabeth Hospital, Birmingham B15 2GW, United Kingdom;; ^n^Institute of Neuroscience and Psychology, University of Glasgow, Glasgow G12 8QQ, United Kingdom;; ^o^Department of Experimental Psychology, University of Oxford, Oxford OX2 6GG, United Kingdom;; ^p^Oxford Centre for Human Brain Activity, Wellcome Centre for Integrative Neuroimaging, Department of Psychiatry, University of Oxford, Oxford OX3 7JX, United Kingdom

**Keywords:** memory, recall, intracranial EEG, hippocampus, gamma power

## Abstract

Adaptive memory recall requires a rapid and flexible switch from external perceptual reminders to internal mnemonic representations. However, owing to the limited temporal or spatial resolution of brain imaging modalities used in isolation, the hippocampal–cortical dynamics supporting this process remain unknown. We thus employed an object-scene cued recall paradigm across two studies, including intracranial electroencephalography (iEEG) and high-density scalp EEG. First, a sustained increase in hippocampal high gamma power (55 to 110 Hz) emerged 500 ms after cue onset and distinguished successful vs. unsuccessful recall. This increase in gamma power for successful recall was followed by a decrease in hippocampal alpha power (8 to 12 Hz). Intriguingly, the hippocampal gamma power increase marked the moment at which extrahippocampal activation patterns shifted from perceptual cue toward mnemonic target representations. In parallel, source-localized EEG alpha power revealed that the recall signal progresses from hippocampus to posterior parietal cortex and then to medial prefrontal cortex. Together, these results identify the hippocampus as the switchboard between perception and memory and elucidate the ensuing hippocampal–cortical dynamics supporting the recall process.

## Significance

How do we adaptively switch from perceiving the external world to retrieving goal-relevant internal memories? To tackle this question, we used—in a cued-recall paradigm—direct intracranial recordings from the human hippocampus complemented by high-density scalp electroencephalography (EEG). We found that a hippocampal signal ∼500 ms after a perceptual cue marks the conversion from external (perceptual) to internal (mnemonic) representations. This sets in motion a recall cascade involving posterior parietal and medial prefrontal cortex, revealed via source-localized and time-resolved EEG alpha power. Together, these results unveil the hippocampal–cortical dynamics supporting rapid and flexible memory recall.

Imagine spotting a familiar face at a (real) conference. As your acquaintance approaches, you frantically try to recall the last time the two of you met and—without sneakily glancing at the nametag—remember what their name was. This example illustrates how adaptive behavior often requires us to shift our focus from external sensory information to internal mnemonic representations. In experimental terms, this scenario constitutes a cued recall task, where a reminder cue may or may not trigger recall of associated mnemonic target information. How does our brain accomplish the feat of converting an external reminder into a target memory?

According to computational models, the hippocampus links disparate cortical representations into a coherent memory trace (1, 2). It retains pointers to the cortical sites involved in the initial experience (3, 4) such that presenting a partial reminder prompts reinstatement of the entire association via hippocampal pattern completion (5, 6). In support of these models, human functional MRI (fMRI) studies linked hippocampal activation with cortical reinstatement of mnemonic target representations during successful recall (7–13). However, the relatively poor temporal resolution of the fMRI signal leaves open whether the hippocampus precedes or follows mnemonic reinstatement, let alone whether hippocampal engagement would mark the rapid switch from perceptual cue to mnemonic target representations.

Moreover, the cognitive complexity and representational richness of memory recall likely requires concerted engagement of wider brain networks (14, 15). Indeed, beyond the hippocampus, neuroimaging work has consistently implicated a particular set of cortical regions in episodic memory tasks (16, 17), herein referred to as the “cortical retrieval network” (CRN). The CRN overlaps with the “default mode network” (18) and includes posterior parietal regions as well as medial prefrontal cortex. It has been linked to retrieval success across multiple stimulus domains (19) as well as to episodic (re)construction processes (20, 21). Critically, a recent study employing “lesion network mapping” suggests that the hippocampus serves as a functional hub linking these cortical nodes in service of memory processes (22). While these results indicate that successful memory relies on intricate hippocampal–cortical interactions, the temporal dynamics within the CRN are challenging to resolve with fMRI alone, hampering understanding of different CRN regions’ contributions (16).

To overcome these limitations, we used intracranial electroencephalography (iEEG) complemented by high-density scalp EEG to reveal 1) the role of the hippocampus in the conversion of perceptual cues to mnemonic targets and 2) the ensuing dynamics in the frontoparietal retrieval network.

## Results

### Behavior.

**Fig. 1.**
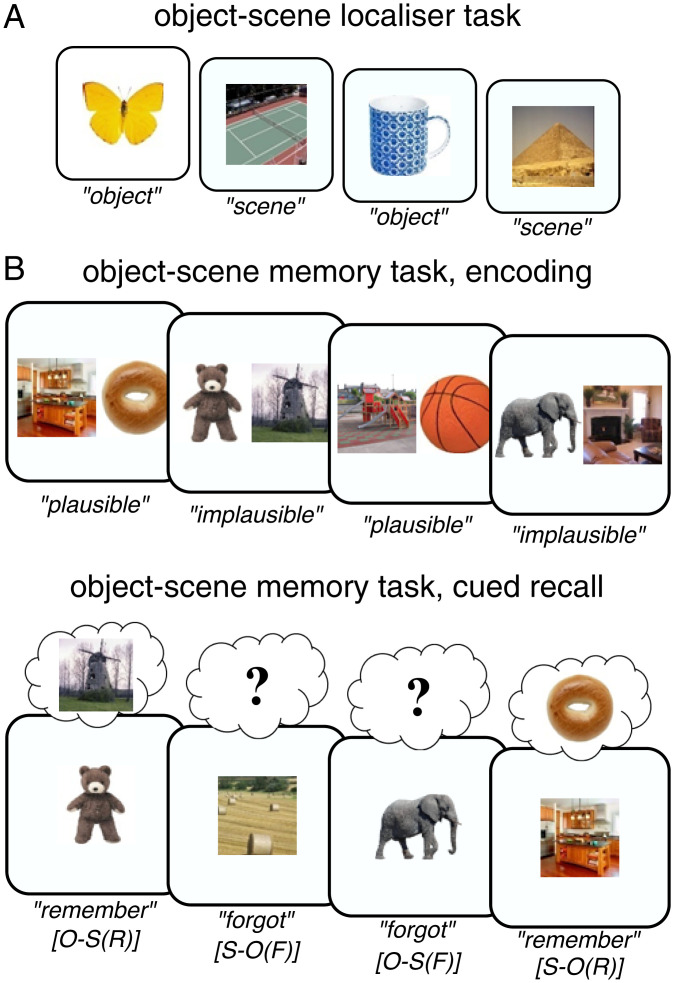
Experimental paradigm. (*A*) In a perceptual localizer session, participants saw trial-unique images of objects and scenes and indicated the category of the given image. This part served as an independent training dataset for multivariate pattern analyses. (*B*) The main experiment employed an object–scene memory task, consisting of an encoding phase (*Top*) and a cued recall phase (*Bottom*). During encoding, participants saw trial-unique object–scene pairs and indicated whether the given combination was plausible or implausible. During cued recall, participants were given either the object or the scene image as the cue and were asked to recall the paired target (scene or object image, respectively). The key conditions were 1) trials in which participants indicated they did remember the target image (“Remember” trials) and 2) trials in which participants indicated they did not remember the target image (“Forgot” trials). Labels below denote the cue-target (memory) status of trials. O = object, S = scene, R = remember, F = forgot.

We used the same memory paradigm (Fig. 1) in an iEEG study (*n* = 11) and a high-density scalp EEG study (*n* = 20). In addition, we conducted “localizer” runs (Fig. 1*A*) to train a classifier to distinguish brain patterns of object vs. scene representations (see below). In the memory experiment (Fig. 1*B*), participants were presented with pairs of object and scene images during encoding. During retrieval, a cued recall task was employed in which only one of the images was shown (“cue”), with the question whether the associated image (“target”) was also remembered. Catch trials were interspersed in which participants were prompted to describe the target image after giving a “Remember” response.

In the iEEG study, accuracy on the localizer task was on average 95% (SEM = 2%) correct (mean reaction time [RT] = 1.40 s, SEM = 0.21). During the cued recall task, iEEG participants indicated they remembered the target on 67% of trials (SEM = 5%). During catch trials, accuracy was 94% (SEM = 2%). RTs were faster for “Remember” trials (mean [M] = 2.59 s, SEM = 0.26) than for “Forgot” trials [M = 5.95 s, SEM = 0.40; *t* (10) = 8.46, *P* < 0.001]. “Remember” RTs did not differ significantly for object vs. scene targets [*t* (10) = 0.40, *P* = 0.695].

In the scalp EEG study, participants remembered 60% (SEM = 3%) of target images. Accuracy on catch trials was 92% (SEM = 2%). RTs were faster for “Remember” trials (M = 1.61 s, SEM = 0.08) than for “Forgot” trials [M = 2.37 s, SEM = 0.17; *t* (19) = 5.30, *P* < 0.001]. Again, RTs did not differ significantly for object vs. scene targets [*t* (19) = 0.73, *P* = 0.476]. Given average RTs for “Remember” trials, iEEG and scalp EEG data were analyzed from −0.5 s to 2.6 s and 1.6 s, respectively.

### A Hippocampal Recall Signal at ∼500 ms.

**Fig. 2.**
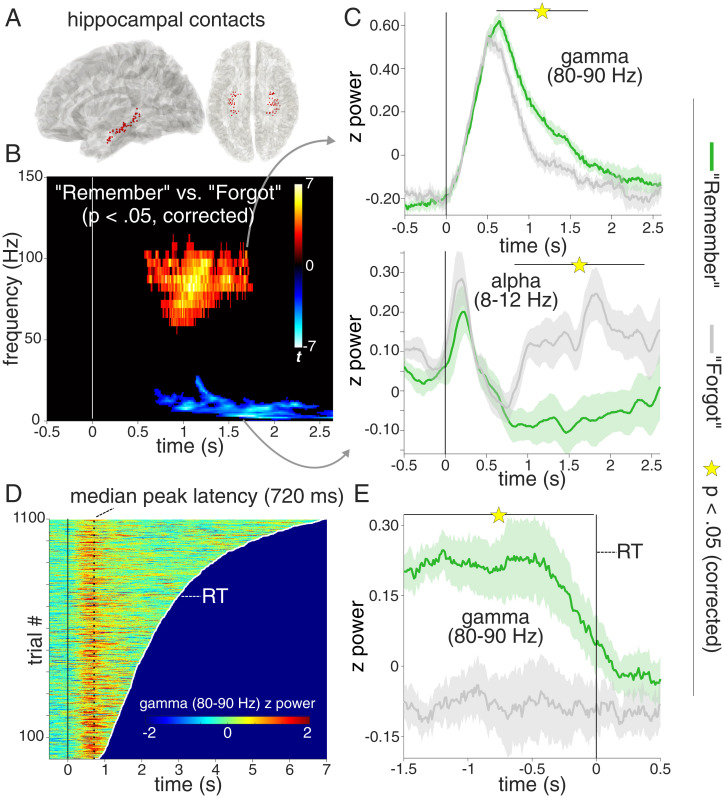
Hippocampal recall effects. (*A*) Hippocampal contacts across participants shown on a normalized sagittal (*Left*) and horizontal (*Right*) brain template. (*B*) Results from a time-frequency analysis (*P* < 0.05, corrected), contrasting “Remember” vs. “Forgot” trials and revealing a cluster in the high gamma range (55 to 110 Hz, peak at 85 Hz) with power increases for “Remember” trials, followed by a cluster in the alpha band (2 to 29 Hz, peak at 10 Hz) with power decreases for “Remember” trials. For an unthresholded map, see *SI Appendix*, Fig. S2*A*. (*C*) Power time courses for “Remember” (green) and “Forgot” (gray) trials in the gamma (*Top*, significant from 0.61 to 1.71 s) and alpha (*Bottom*, significant from 0.84 to 2.41 s) ranges. Lines show condition means ± SEM of condition differences across participants. For time courses encompassing the entire cluster frequency range, see *SI Appendix*, Fig. S2*B*. (*D*) Hippocampal gamma power (80 to 90 Hz) across time from −0.5 s to RT across all “Remember” trials (pooled across participants), sorted based on trial-specific RT (white line). Dashed vertical line indicates median peak latency across all trials (720 ms). For an RT-locked representation, see *SI Appendix*, Fig. S2*D*. (*E*) Response-locked hippocampal gamma power (80 to 90 Hz) for “Remember” (green) and “Forgot” (gray) trials, significant from −1.5 to −0.020 s (*P* < 0.05). Lines show condition means ± SEM of condition differences across participants.

Our first analysis examined spectral power in the hippocampus (Fig. 2*A*) during successful vs. unsuccessful cued recall (“Remember” vs. “Forgot”). As shown in Fig. 2*B*, we observed an extended cluster in the gamma frequency range (55 to 110 Hz, 570 to 1,730 ms, peak frequency: 85 Hz) in which “Remember” trials elicited greater power than “Forgot” trials [P_cluster_ = 0.011, summed cluster *t* (10) = 2,434]. The gamma effect was followed by a power decrease for “Remember” trials relative to “Forgot” trials below 30 Hz, with a distinctive peak in the alpha band [2 to 29 Hz, 680 to 2,600 ms, peak frequency: 10 Hz; P_cluster_ = 0.004, summed cluster *t* (10) = −6,391]. These hippocampal gamma/alpha effects were highly reliable across participants (11/11) and across subselections of hippocampal contacts (anterior hippocampus, posterior hippocampus, or nonpathological tissue according to clinical diagnostics; *SI Appendix*, Fig. S1). Hippocampal gamma and alpha power time courses are shown in Fig. 2*C*, averaged across the peak ranges of 80 to 90 Hz for gamma and 8 to 12 Hz for alpha (note though that results remain the same when using a weighted average across the entire clusters of significant frequencies; *SI Appendix*, Fig. S2). Across participants, there was a significant negative correlation between the earlier gamma effect (80 to 90 Hz, averaged from 500 to 1,500 ms) and the later alpha effect (8 to 12 Hz, averaged from 1,500 to 2,500 ms) [Pearson *r* (11) = −0.71, *P* = 0.015; see *SI Appendix*, Fig. S2*C* for a two-dimensional correlation map]. This finding replicates a previous report in which we found a gamma power increase followed by an alpha power decrease for successful vs. unsuccessful associative recognition memory (23) and extends it to a cued recall paradigm.

The hippocampal recall effect between ∼500 and 1,500 ms could emerge from two different scenarios. First, it could represent a “hard-wired” peak reflecting input propagation delays from visual cortex (24), followed by sustained engagement from 500 to 1,500 ms that accompanies the recall process. Alternatively, it could emerge from transient events [e.g., discrete bursts (25)] occurring at different latencies across trials, with gamma peak latencies perhaps tracking RTs. To adjudicate between these alternatives, we first plotted hippocampal gamma power (80 to 90 Hz) for all “Remember” trials as a function of RT. As shown in Fig. 2*D*, this revealed a highly consistent peak at ∼700 ms after cue onset, regardless of RT. Next, to test whether the hippocampal gamma effect accompanies successful recall in a sustained fashion, we plotted response-locked gamma power. As shown in Fig. 2*E*, this revealed that the gamma effect coterminated with a participant’s “Remember” response [significant cluster from −1,500 ms to −20 ms, summed cluster *t* (10) = 748, P_cluster_ = 0.001]. Together, these results suggest that a hippocampal recall signal sets in at ∼500 ms and sustains until retrieval is complete (see *Discussion*).

### From Perception to Memory via the Hippocampus.

**Fig. 3.**
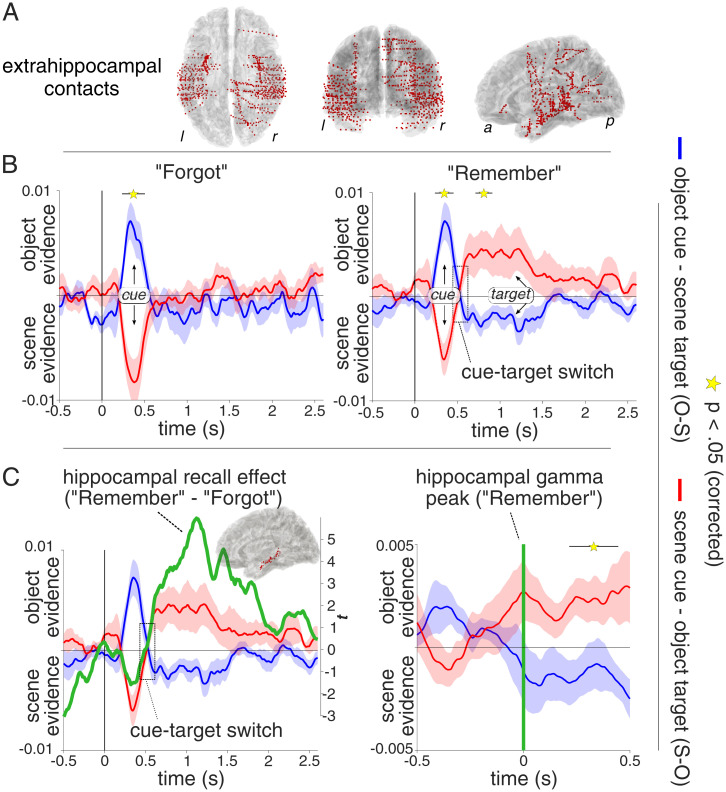
Switch from cue to target representations. (*A*) Extrahippocampal contacts across participants shown on a normalized horizontal (*Left*), coronal (*Middle*), and sagittal (*Right*) brain template. (*B*) Object and scene evidence, based on a classifier trained on the “localizer” session, for O-S (blue) and S-O (red) trials, separately for “Forgot” (*Left*) and “Remember” (*Right*) trials (mean ± sem across participants). Within the first 500 ms, classifier evidence reflects the cue category. Only for “Remember” trials, classifier evidence then switches (dashed rectangle) to reflect the recalled target category. (*C*, *Left*) Overlay of the hippocampal recall effect (“Remember” vs. “Forgot” gamma *t* statistic, smoothed with a 100-ms sliding window) onto the extrahippocampal switch from cue to target representation for “Remember” trials. (*Inset*) Hippocampal contacts across patients. (*C*, *Right*) Object and scene evidence for “Remember” trials as in *B*, *Right*, but realigned to trial-by-trial hippocampal gamma peaks (time 0, vertical green line). Stars denote significant differences of classifier evidence for O-S trials vs. S-O trials (*P* < 0.05, corrected).

To investigate whether the hippocampal recall signal marks the switch from perceptual to mnemonic representations, we used participants’ extrahippocampal iEEG contacts (Fig. 3*A*) to train a linear classifier on object vs. scene trials during the separate localizer sessions. First examining cross-validated within-localizer performance, results confirmed high classification accuracy (*SI Appendix*, Fig. S3), with peak performance between 300 to 400 ms after stimulus onset. To capture object and scene representations during recall, a classifier was then trained on the 300- to 400-ms localizer window and applied to retrieval, yielding a time series of continuous object vs. scene evidence for successful and unsuccessful object cue–scene target (O-S) and “scene cue–object target” (S-O) retrieval trials (Fig. 1). Note that class labels were coded such that positive classifier values denote object evidence and negative classifier values denote scene evidence. During O-S trials, positive values thus signify cue (object) evidence, whereas negative values signify target (scene) evidence. Conversely, during S-O trials, positive values signify target (object) evidence, whereas negative values signify cue (scene) evidence.

As shown in Fig. 3*B*, classifier evidence was, as expected, strongly cue-driven (i.e., object evidence for O-S trials and scene evidence for S-O trials) within the first 500 ms for both “Forgot” and “Remember” trials [O-S(F) vs. S-O(F): P_cluster_ = 0.002, 245 to 495 ms, summed cluster *t* (10) = 185; O-S(R) vs. S-O(R): P_cluster_ = 0.005, 245 to 445 ms, summed cluster *t* (10) = 158]. Critically, only “Remember” trials then showed evidence for a representational switch from the cue to the target category. Specifically, for O-S(R) trials, representational patterns shifted from object (cue) to scene (target) evidence at ∼500 ms and analogous for S-O(R) trials, i.e., shifting from scene (cue) to object (target) evidence. The difference in scene evidence for O-S(R) and object evidence for S-O(R) was significant from 720 to 900 ms [P_cluster_ = 0.016, summed cluster *t* (10) = 95]. Target evidence (i.e., scene evidence for O-S trials and object evidence for S-O trials) from 720 to 900 ms was significantly greater than chance for both for S-O(R) trials [*t* (10) = 2.37, *P* = 0.040] and O-S(R) trials [*t* (10) = −2.70, *P* = 0.022], without a significant difference between the two [*t* (10) = 1.57, *P* = 0.148]. Moreover, the combined target evidence (i.e., scene evidence for O-S trials and object evidence for S-O trials) was significantly greater for “Remember” trials than “Forgot” trials from 555 to 895 ms [P_cluster_ = 0.003, summed cluster *t* (10) = 217]. Together, these results show that during successful cued recall extrahippocampal activation patterns dynamically switch from representing the perceived cue category to representing the retrieved target category.

Inspection of gamma power (Fig. 2*C*) and classification time courses (Fig. 3*B*) raises the intriguing possibility that the hippocampal gamma power increase during “Remember” trials at ∼500 ms marks the moment at which the brain switches from cue to target representations. Fig. 3*C*, *Left* shows the hippocampal gamma recall effect (80 to 90 Hz, *t* statistic of “Remember” vs. “Forgot”) superimposed on the classification effect, suggesting that these two effects indeed evolve in tandem. Across participants, the fractional area latencies (26) (50% of cumulative positive values from 0 to 1,500 ms) of 1) the hippocampal gamma effect (“Remember” vs. “Forgot”) and 2) target category evidence during “Remember” vs. “Forgot” trials showed a trend for a positive correlation [*r* (11) = 0.52, *P* = 0.098].

To assess the link between hippocampal gamma and the representational cue–target switch more directly, we repeated the classification analysis for “Remember” trials but realigned each trial to its hippocampal gamma power peak. Fig. 3*C*, *Right* illustrates the switch from cue to target evidence around the hippocampal gamma peak. Note that the hippocampal recall effect sets in ∼200 ms prior to the actual gamma peak, consistent with the notion that the representational cue–target switch starts as hippocampal gamma power distinguishes “Remember” from “Forgot” trials. Comparison of hippocampal gamma-peak locked classifier evidence for O-S(R) vs. S-O(R) trials across time revealed a significant difference from 215 to 445 ms after the gamma peak [P_cluster_ = 0.013, summed cluster *t* (10) = 120]. Note that the effect size of target evidence [sum of significant *t* values, O-S(R) vs. S-O(R)] was larger in the hippocampal-peak-locked than cue-onset-locked analysis (120 vs. 95, 26% increase).

To further quantify the representational switch around hippocampal gamma peaks, we averaged classifier evidence for O-S(R) and S-O(R) trials across a 500-ms pre- and a 500-ms post-hippocampal gamma peak window (Fig. 3*C*, *Right*) and conducted a repeated-measures ANOVA including the factors Time Window (pre, post) and Trial Type (O-S, S-O). Results revealed a significant interaction [*F*(1,20) = 12.18, *P* = 0.006] in the absence of any main effect (both *P* > 0.22). As a control analysis, we repeated the peak-locked analysis but aligning classifier data to trialwise negative peaks in alpha power (8 to 12 Hz). No main effect of Time Window, Trial Type, or Time Window × Trial Type interaction was observed (all *P* > 0.11). In fact, there was a significant three-way interaction of Time Window × Trial Type with Frequency Band (gamma, alpha) [*F*(1,10) = 8.03, *P* = 0.018], corroborating that the representational switch from cue to target evidence was specific to hippocampal gamma peaks.

### Recall Signals across the CRN.

Results from our iEEG sample showed that ∼500 ms after cue onset a hippocampal signal distinguishes successful from unsuccessful cued recall and marks the switch from cue to target representations. However, hippocampal engagement alone is unlikely to be sufficient for full-blown memory recall. To elucidate the cortical dynamics associated with cued recall, we conducted the same experiment in a sample of 20 healthy participants using high-density scalp EEG.

**Fig. 4.**
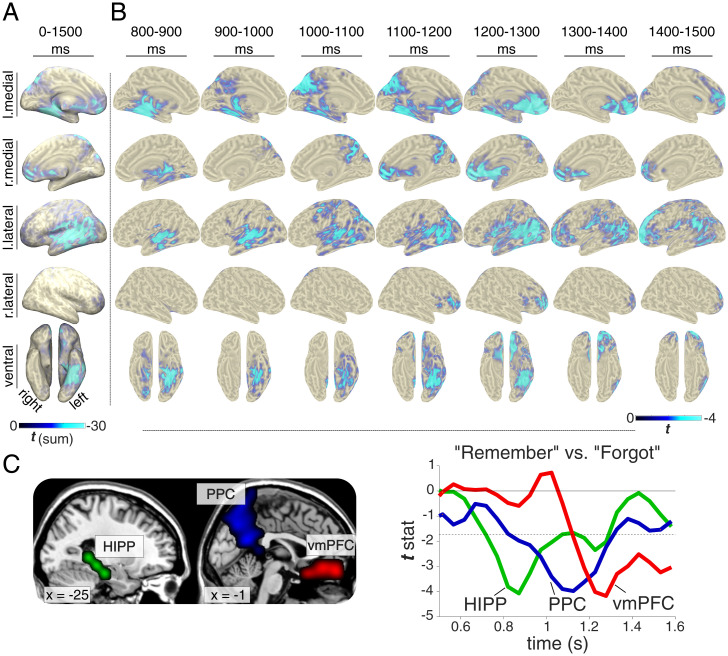
Brain-wide recall dynamics. Results from source-localized EEG alpha (8 to 12 Hz) power, comparing “Remember” vs. “Forgot” trials. (*A*) Voxel × time results (P_cluster_ < 0.05), revealing recall effects across the core retrieval network (including hippocampus, PPC, LTC, and vmPFC). Color reflects sum of significant *t* values across time. (*B*) Time-resolved results (proceeding in 100-ms steps), showing only time windows with significant effects (each map thresholded at P_cluster_ < 0.05). (*C*, *Left*) Regions of interest defined as the overlap of significant voxels resulting from the main voxel × time analysis (cf. *A*) and AAL masks for hippocampus (HIPP), medial PPC, and vmPFC. *x* coordinates refer to MNI space. (*C*, *Right*) *t* values for the comparison of “Remember” vs. “Forgot” from 0.5 to 1.6 s in each region. Dashed horizontal line marks the threshold for *P* < 0.05 (uncorrected).

A first sensor × frequency × time comparison of “Remember” vs. “Forgot” revealed an extended cluster of relative power decreases for “Remember” trials (*SI Appendix*, Fig. S4), with a peak effect at 9 Hz (similar to the iEEG study; cf. Fig. 2*B*). We next projected the sensor EEG data into source space (see *Materials and Methods*), extracted alpha power (8 to 12 Hz) in the resulting virtual voxels, and examined how the recall signal (alpha power decrease for “Remember” compared to “Forgot” trials) evolves across time. Performing the analysis across the entire 0- to 1.500-ms interval revealed significant recall effects across the CRN, including medial temporal lobe (MTL), posterior parietal cortex (PPC), lateral temporal cortex (LTC) and ventromedial prefrontal cortex (vmPFC; Fig. 4*A*). This result replicates and extends a previous study in which we used source-localized magnetoencephalography (MEG) alpha power to reveal recall effects across the CRN (27).

Critically, we next performed the same analysis in a time-resolved fashion, progressing from 0 to 1,500 ms in 100-ms steps, each step averaging alpha power across a 100-ms time window. Each comparison was again cluster-corrected for multiple comparisons across virtual voxels. As shown in Fig. 4*B*, the first time window to show a significant recall effect occurred from 800 to 900 ms and encompassed the MTL. It is worth pointing out that this is also the time window in which our intracranial data showed an alpha power decrease for “Remember” trials in the hippocampus (Fig. 2*B*), providing a link between the two datasets and imaging modalities. From ∼900 to 1,200 ms the recall effect encompassed medial and lateral PPC, followed by a recall effect in vmPFC from ∼1,100 to 1,500 ms.

To quantify these latency differences statistically, we extracted alpha power (8 to 12 Hz) time courses in voxels showing significant effects in the initial voxel × time contrast (Fig. 4*A*) and overlapping with anatomically defined regions of interest for hippocampus, medial PPC, and vmPFC (Fig. 4*C*). The alpha power effect (“Remember” vs. “Forgot”) was then binned into 100-ms segments from 800 to 1,500 ms and subjected to a repeated-measures ANOVA (Greenhouse–Geisser-corrected) including the factors Region and Time. Results confirmed a significant Region × Time interaction [*F*(4.03, 76.48) = 3.75, *P* = 0.008] in the absence of any main effect (both *P* > 0.47). As illustrated in Fig. 4*B*, this interaction was due to the recall effect first emerging in the hippocampus, followed by PPC and then vmPFC. Together, these results reveal a hitherto unknown sequence of recall signals within the CRN, starting in the MTL and progressing via PPC to vmPFC.

## Discussion

Our study elucidates the role of the hippocampus as a switchboard from perception to memory and unveils the ensuing cortical dynamics supporting the recall process. Using a simple and robust cued recall paradigm (Fig. 1), iEEG recordings first revealed a hippocampal signal in the high gamma range (55 to 110 Hz) distinguishing between successful and unsuccessful recall from 500 ms onward (Fig. 2*B*). This gamma effect was followed by a relative power decrease for successful recall in the alpha band starting at ∼800 ms, with the two effect sizes correlating across participants. Using multivariate pattern analysis (MVPA), we observed—for successful recall only—a representational switch from the cue stimulus category (<500 ms) to the target stimulus category (>500 ms; Fig. 3*B*). Time-locking the MVPA to hippocampal gamma peaks showed cue evidence before and target evidence after these peaks, suggesting that the hippocampal gamma increase marks the moment at which brain states shift from perceptual to mnemonic representations (Fig. 3*C*). Moving beyond the hippocampus with high-density scalp EEG, we first established engagement of the CRN during successful recall, including PPC and vmPFC (Fig. 4). Critically, using time-resolved alpha power in source space, we found a particular recall cascade across the CRN: Starting at ∼800 ms in the MTL, successful recall subsequently entailed PPC at ∼900 ms, followed by vmPFC at ∼1,100 ms.

### The Hippocampus as the Switchboard from Perceptual Cues to Mnemonic Targets.

Our data provide empirical support for the long-held notion that the hippocampus orchestrates cortical pattern completion (4–6). Owing to modality-specific methodological limitations across species, such empirical evidence has been challenging to obtain. That is, fMRI lacks the temporal resolution to pinpoint a hippocampal signal preceding target reinstatement, although recent analytical advances have yielded some progress in resolving fine-grained memory dynamics with fMRI (12, 28). Scalp EEG and MEG, combined with advanced source reconstruction methods (29, 30), in principle provide adequate levels of spatial and temporal precision to uncover whole-brain memory dynamics (27, 31). However, ambiguities remain when interpreting activation in deeper sources such as the MTL, at least without converging evidence from other imaging modalities. Optogenetic studies in mice have shown that experimental activation of hippocampal cell assemblies elicits contextual fear behavior (32) and that silencing hippocampal cells abolishes reinstatement of memory representations in cortical structures such as entorhinal cortex, perirhinal cortex, and retrosplenial cortex (33). However, it remains open to what extent contextual fear conditioning captures the intricacies of episodic memory recall in humans. Moreover, these studies remain agnostic about the fast temporal relationship between hippocampal and cortical engagement during episodic memory recall. Nevertheless, a similar intervention approach in human recordings (e.g., disrupting hippocampal processing at 500 ms via electrical stimulation) would allow conclusions about the causal nature of the hippocampal effects we observed vis-à-vis pattern completion.

In any case, the hippocampal recall effect (Fig. 2) unifies and extends a series of recent human electrophysiological results. Specifically, a hippocampal recall signal starting at ∼500 ms after onset of a retrieval cue has been reported for evoked field potentials (34), high gamma power (23), and single neuron firing rates (35), attesting to the convergence of these electrophysiological measures (36). It should be noted that while the frequency range of our high-gamma effect (55 to 110 Hz) is in agreement with a number of hippocampal recall effects reported in iEEG studies (37–39) another recent study (40) identified a more narrow-band recall effect in a lower gamma range (40 to 50 Hz), likely reflecting a complementary neural mechanism (41).

Fig. 2, moreover, illustrates the sustained nature of the hippocampal recall effect, extending from ∼500 to 1,500 ms after cue onset. We have interpreted the 500-ms onset as reflecting conduction delays from sensory regions to the hippocampus (24) and the ensuing ∼1-s period as reflecting recurrent hippocampal–cortical interactions in service of memory retrieval (42). However, this pattern could also emerge from transient bursts (43, 44) igniting the recall process at different latencies across trials, perhaps tracking trial-specific RTs. As shown in Fig. 2*D*, our data point to a highly consistent gamma peak occurring at ∼700 ms after cue onset irrespective of RT, corroborating the notion that it reflects a relatively “hard-wired” delay at which a hippocampal recall signal sets in, at least in the experimental context of our cued recall paradigm. This gamma peak latency agrees with previous studies examining single neuron firing latencies in memory-selective hippocampal neurons (35, 45) and event-related potential recordings from the hippocampus (46). Importantly, though, we also found that hippocampal gamma remains sustained until a memory response is given (Fig. 2*E*), suggesting that hippocampal engagement accompanies extrahippocampal reinstatement processes throughout recall. It deserves mention that the consistent onset notwithstanding the sustained recall signal may well include discrete gamma bursts/ripples occurring at different latencies across trials (25, 47).

While we here focused on target reinstatement in extrahippocampal sites, theoretical models implicate a prior pattern completion process within the hippocampus to retrieve the “index” of the target representation (4, 48). In the current paradigm, it is challenging to disentangle whether any similarity between a given retrieval trial and its encoding counterpart would reflect such pattern completion processes or the perceptual match of the cue image with the encoding display (Fig. 1). That said, in a previous study we found that an intrahippocampal pattern completion process commenced ∼500 ms after cue onset (23) and directly correlated with high gamma power increases. Together, these data suggest that at ∼500 ms a cue representation reaches the hippocampus and induces an intrahippocampal pattern completion process. If successful (reflected in increased high gamma power), this ignites sustained reinstatement of the episodic target representation in cortex (42). The subsequent decrease in hippocampal alpha power might then reflect increased levels of information processing, as postulated and shown for cortical information processing (49, 50). As elaborated below, this alpha power decrease subsequently tracks the dynamic recall signal throughout the CRN.

Another pressing question is whether cue/target information is represented in the hippocampus itself. The relatively small number of hippocampal contacts (*SI Appendix*, Table S1) precluded us from directly comparing cue/target decodability in hippocampus vs. cortex, but recent single-neuron recordings revealed both memory-selective and stimulus-selective neurons in the hippocampus (35, 45). Future iEEG studies with sufficient hippocampal coverage could thus examine the temporal dynamics between hippocampal mnemonic/pattern completion signals vis-à-vis hippocampal and cortical stimulus-specific signals.

### Temporal Dynamics in the CRN.

The process of reinstating a full-blown episodic memory and deploying adaptive behavior most likely relies on intricate interactions across multiple cortical areas beyond the hippocampus (14, 16). Apart from content-specific areas involved in reinstatement (11, 13, 51), recent fMRI research has revealed a cortical brain network consistently emerging during successful recall (17). This network includes medial and lateral parietal cortex (PPC) and vmPFC, all of which are densely connected with the hippocampus (22, 52). What is still unresolved, however, is the particular role each of the different CRN nodes play (16).

Capitalizing on the temporal resolution of EEG, we found that within the CRN a recall effect (alpha power decreases) first emerged in the MTL, followed by medial PPC and finally vmPFC (Fig. 4). Involvement of the hippocampus in this recall task is corroborated by our intracranial data revealing a hippocampal alpha power effect spanning the same time and frequency window (Fig. 2) as well as by a previous fMRI study using the same paradigm (12). This result adds to recent evidence emphasizing the feasibility of using source-localized M/EEG recordings to examine hippocampal memory processes (27, 53–55).

In any case, the functional significance of the MTL–PPC–vmPFC trajectory is unknown at present. There is ongoing debate about the role of different PPC regions (e.g., posterior midline, superior parietal lobule, inferior parietal lobule) in episodic retrieval (56, 57), but one prevalent view is that involvement of PPC regions scale with the amount of mnemonic evidence (57). Likewise, the functional parcellation and the specific role of medial PFC in memory retrieval is still poorly understood (58), although there is consensus about a role of prefrontal areas in higher-order information integration and action planning (59, 60). One tentative scenario could thus be that PPC serves as an “episodic buffer” (61), accumulating episodic details that are reinstated in content-specific areas through hippocampal pattern completion. Ventromedial PFC might then integrate this mnemonic evidence with the current task set and initiate goal-directed behavior. On that note, we do not mean to imply that the process of memory retrieval per se starts in the hippocampus—our analyses merely suggest that the hippocampus is where the difference between successful and unsuccessful recall first emerges. Apart from early perceptual regions processing the cue information, strategic recall likely requires top-down input from prefrontal control sites. For instance, a recent iEEG study employing simultaneous PFC and hippocampal recordings (62) reported a driving influence from PFC to hippocampus in a directed forgetting paradigm (interestingly with forgetting being accompanied by an increase in hippocampal alpha power).

In sum, our study suggests that a high-gamma signal in the hippocampus mediates the conversion from perceptual cues to mnemonic targets during successful recall. The ensuing decrease in alpha power then propagates from the hippocampus throughout the CRN, perhaps reflecting the accumulation of mnemonic target evidence and preparation of goal-directed behavior.

## Materials and Methods

### Participants.

For the iEEG study, 10 patients from the Queen Elizabeth Hospital in Birmingham and 1 patient from La Paz University Hospital in Madrid, all suffering from medically intractable epilepsy, volunteered (6 male, 5 female, aged 24 to 53 y, M = 34.45). Additional patient characteristics are listed in *SI Appendix*, Table S1. Ethical approvals were granted by the National Research Ethics Service UK (code 15/EM/0182) and by the Clinical Research Ethics Committee at La Paz University Hospital Madrid (code IP-2401), respectively.

Twenty healthy, right-handed participants (12 male, 8 female) with normal or corrected-to-normal vision volunteered in the EEG experiment. They were aged 20 to 33 y (M = 25.01). An additional six participants had been rejected from analysis due to noisy EEG data (*n* = 2), inconsistent Polhemus data (*n* = 2), or poor memory performance (<40% “Remember” trials, *n* = 2). All participants were fluent English speakers. Participants gave written informed consent and received course credits or financial remuneration. Ethical approval was granted by the University of Birmingham Research Ethics Committee (ERN_14-1379). In additional to functional recordings, structural MRIs were acquired for 15 participants.

### EEG Experimental Procedure.

The stimulus material consisted of 712 color images sized 200 × 200 pixels, half depicting objects and half depicting scenes. It was based on a set of images used in previous studies (12, 63) supplemented with additional images obtained via a Google search that matched the main image set in style. Participants received written and verbal instructions.

Before and after the main experiment, participants performed “localizer” runs. In each run, participants completed 10 practice trials (5 objects, 5 scenes) that were not recorded followed by 100 unique images (50 objects, 50 scenes) presented in the center of the screen. Each trial started with a fixation cross presented for 1.5 ± 0.1 s. Subsequently, an object or scene image was superimposed on the fixation cross. Participants had to press a button to indicate whether the image depicts an object or a scene. After 1 s, a legend appeared at the bottom of the screen reminding participants of the assignment between left/right buttons and object/scene. To avoid contamination of the classifier by response mapping, this assignment was flipped in the second localizer run (initial assignment counterbalanced across participants). The trial terminated after a button press, although the image was shown for a minimum of 2 s and a maximum of 10 s. The localizer was included in the EEG study to match the iEEG paradigm, but data are not used in the present paper.

The main experiment consisted of eight runs following the paradigm used in ref. 12. Each run was split into four blocks: a preencoding delay block, an encoding block, a postencoding delay block, and a retrieval block. Before and after each block, a progress bar was displayed for 6 s, alerting participants to the impending start of the next block. During delay blocks, random numbers between 0 and 100 were shown, and participants pressed the left key for even numbers and the right key for odd numbers. This phase was self-paced, with a new number appearing immediately after a button press. Participants were encouraged to perform the task as fast as possible while maintaining high performance. Each delay block lasted 3 min.

Each encoding block consisted of 32 trials. Each trial started with a fixation cross presented for 1.5 ± 0.1 s. Subsequently, a unique, randomly chosen object–scene pair was shown. During 16 randomly assigned trials, the object appeared left of the center and the scene appeared right, with the opposite arrangement for the other 16 trials. The object-scene pair remained on the screen until a button was pressed, but it was displayed for a minimum of 2.5 s and a maximum of 4 s. Participants used their right hand and indicated with the index finger that the object-scene pair was “plausible”, i.e., likely to appear in real life or nature, or used their middle finger to indicate that it was “implausible.”

Each retrieval block comprised 32 trials. Each trial commenced again with a fixation cross 1.5 ± 0.1 s. Subsequently, a cue was shown in the center of the screen, either an object or a scene taken from the previous encoding block. The object–scene pair remained on the screen until a button was pressed, but it was displayed for a minimum of 2.5 s and a maximum of 6 s. Participants were asked to indicate whether they “remember” (index finger) or “forgot” (middle finger) the corresponding paired associate. Half of the cues were objects, the other half scenes. Across the 32 trials, each cue type (object or scene) was presented in miniblocks of eight consecutive trials alternating between eight object cues (O) and eight scene cues (S), i.e., O-S-O-S or S-O-S-O. Participants were instructed to only press “Remember” when their memory was vivid enough to give a detailed description of the associate. To ensure that this is indeed the case, in 20% of the cases “Remember” responses were followed by the instruction to enter a description of the target associate using the computer keyboard (“catch trials”). The experiment was programmed with Psychophysics Toolbox Version 3 (64).

### iEEG Experimental Procedure.

For the iEEG study, the procedure was largely similar, with a few modifications. The stimulus pool for the memory portion consisted of 192 objects and 192 scenes (drawn from the same pool as described above). To accommodate different levels of cognitive capacity across epilepsy patients, we prepared three versions of the experiment, varying in the duration of each run. In Level 1, an encoding/retrieval block consisted of eight trials, resulting in a total of 24 runs including a pre/postencoding delay of 30 s. In Level 2, there were 12 runs with 16 trials per encoding/retrieval block and 60 s delay periods. In Level 3, there were six runs with 32 trials per encoding/retrieval block and 120-s delay periods. Which version was used depended on performance on a short practice run at difficulty Level 1. Two patients performed the task at Level 1, four at Level 2, and the remaining five patients at Level 3. In terms of stimulus timing, responses were self-paced, but images remained on the screen for a minimum of 2 s and a maximum of 10 s (at which point the response was coded as “invalid” and included in the “Forgot” condition). Instead of typing in responses during the ∼20% catch trials, patients verbally described the paired associate and responses were transcribed by the experimenter. In order not to overtax patients, runs were spread across one to three experimental sessions, with an effort to keep sessions close in time. Again, object/scene localizer runs were conducted before and after each memory session. The same set of 50 object and 50 scene images was used repeatedly, images and response legend remained on the screen for a minimum of 2 s and a maximum of 10 s, and no switch of response finger assignment was introduced.

### iEEG Acquisition and Preprocessing.

iEEG was recorded for presurgical epilepsy diagnosis using laterally implanted depth electrodes. Electrode shafts contained 5 to 15 contacts. Data were digitized at 512 Hz (*n* = 1) or 1,024 Hz (*n* = 10). Intra- and extrahippocampal contacts were identified based on the postimplantation structural MRI. Contacts with hardware artifacts or contamination with clear epileptiform background activity were discarded based on visual inspection (average of 6% across patients). The numbers of contacts per patient included in the analyses are listed in *SI Appendix*, Table S1. For hippocampal contacts, data were locally rereferenced to a white-matter contact on the same electrode. For extrahippocampal contacts, a common median reference including all contacts was used to rereference the data.

### EEG Acquisition and Preprocessing.

EEG was recorded with 128 sintered Ag/AgCl active electrodes and a BioSemi Active-Two amplifier. The signal was digitized at a rate of 1,024 Hz on a second computer via ActiView recording software (BioSemi). Electrode positions and headshape were measured using a Polhemus FASTRAK device in conjunction with Brainstorm (65). All EEG data processing was performed in MATLAB using FieldTrip (66). Data were down-sampled to 256 Hz, high-pass-filtered at 0.1 Hz using a windowed sinc FIR filter and low-pass-filtered at 100 Hz using a Butterworth IIR filter. Furthermore, a band-stop filter was applied at 50 Hz to remove line noise. Retrieval data were then segmented into epochs, starting at −1 s and ending at the time of the button press +1 s, or at 6 s poststimulus, whichever was shorter. Noisy EEG channels were identified by visual inspection and discarded. Subsequently, Infomax ICA (67) was used to clean the data. To this end, all epochs were manually inspected and artifact trials containing muscle artifacts or mechanical artifacts (≈10%) were discarded. The resultant data were high-pass-filtered above 1 Hz and ICA was applied. Using visual inspection of the spatial patterns, time-series and power spectra, ICA components associated with eye blinks, eye movements, and electromyography were rejected from the original data (prior to manual artifact rejection and high-pass filtering). Next, discarded EEG channels that were interpolated using a weighted average of the neighboring channels. Finally, the data were rereferenced using a common average reference.

### EEG Source Modeling.

Individual structural MRIs were segmented into gray matter, white matter, cerebrospinal fluid, skull, and scalp compartments. For five participants, individual MRIs were not available and the standard Montreal Neurological Institute (MNI) template was used instead. As geometric model of the head, a hexahedral mesh with a shift of 0.3 was used. The FieldTrip-SimBio pipeline (68) was used with tissue conductivities of 0.33, 0.14, 1.79, 0.01, and 0.43 in order to create a finite element method volume conduction model. To coregister the MRI with the Polhemus coordinates of the electrodes, the fiducials (nasion, left pre-auricular, right pre-auricular) were manually identified in each MRI. A source grid model with 10-mm spacing and 3,294 grid points was defined in MNI space and mapped onto each participant’s MRI. This ensured that a given grid point corresponded to the same anatomical location across participants.

The data were projected into source space using linearly constrained minimum-variance beamformers (69). Retrieval trials were band-pass-filtered in the 2- to 30-Hz band and trialwise covariance matrices were averaged and regularized.

For region-of-interest-based analyses in source space, we used anatomical masks provided by the Automated Anatomical Labeling (AAL) atlas (70). The following bilateral masks were included: Hippocampus: 'Hippocampus'; PPC: 'Precuneus', 'Cingulum_Post'; vmPFC: 'Frontal_Med_Orb', 'Frontal_Sup_Orb', 'Rectus'.

### Time-Frequency Analysis.

For both iEEG and EEG, short-time Fourier analysis of the retrieval data were performed using FieldTrip with sliding time windows in 10-ms (iEEG) or 25-ms (EEG) steps. For a lower frequency range (2 to 29 Hz iEEG, 2 to 48 EEG, 1-Hz steps), the window length was set to five cycles of a given frequency and the windowed data segments were multiplied with a Hanning taper. For calculation of EEG alpha power (8 to 12 Hz) in source space, 50-ms time steps were used. For iEEG hippocampal gamma power (30 to 150 Hz, 5-Hz steps, iEEG only), we applied multitapering using a fixed window length of 400 ms and seven orthogonal Slepian tapers. To remove outliers, the 10% most extreme power values across trials (within each condition separately) were discarded within each contact and for each time/frequency bin. Remaining power values for each frequency and contact were normalized via z-transformation across all time points (including both conditions), including a 500-ms prestimulus baseline interval. Z power time series were then averaged across hippocampal contacts. Analyses were restricted to 2.6-s postcue onset for iEEG data and to 1.5-s postcue onset for EEG data, as this marked the respective average response time for “Remember” trials across participants (leaving a 100-ms buffer in the EEG data to avoid contamination by the motor response).

### Multivariate Analysis.

Prior to classification, both localizer and retrieval data were preprocessed as follows: Z-scoring was applied across trials for each time point separately to normalize channel variances and remove baseline shifts. Z-scoring was first done across trials within each run in order to account for signal changes across time. The runs were then concatenated and jointly normalized using another z-scoring operation. To mitigate high-frequency noise, temporal smoothing was applied to the raw EEG voltages via a 100-ms moving average window.

Classification of iEEG data was then performed using MVPA-Light (71). For all multivariate analyses, a regularized linear discriminant analysis (LDA) (72) was trained to differentiate between object and scene trials. Formally, LDA is characterized by a weight vector $w\in \;{\mathbb{R}}^{P}$ (*P* = number of extrahippocampal contacts) that represents the normal to the hyperplane and bias b∈ ℝ that represents the classification threshold. For two classes, these parameters are given by

$$w=\Sigma^{-1}\left(\mu_{\mathrm{object}}-\mu_{\mathrm{scene}}\right)$$

$$b\;=\;-\frac{1}{2}\;w^{⊤}\;\left(\mu_{\mathrm{object}}+\mu_{\mathrm{scene}}\right),$$

where $\Sigma \in \;{\mathbb{R}}^{P\times P}$ is the pooled covariance matrix and $\mu_{\mathrm{object}}$ and $\mu_{\mathrm{scene}}$ are the mean voltages across object and scene trials, respectively. To increase the robustness of the model for small sample sizes, shrinkage regularization was used (73). The empirical covariance Σ matrix was replaced by a convex combination of Σ and the identity matrix I scaled by a factor ν such that trace(νI) = trace(Σ),

$$\tilde{\Sigma }=\left(1-\lambda \right)\Sigma +\lambda \nu I.$$

The optimal value for the regularization hyperparameter λ∈[0,1] was estimated using the Ledoit–Wolf approach (74). When applied to a test sample $x\in \;{\mathbb{R}}^{P}$, the classifier produces a decision value (dval) that represents the signed distance to the hyperplane,

$$\mathrm{dval}\;=\;w^{⊤}x\;+\;b.$$

Here, a positive dval is evidence for the presence of a brain pattern associated with an object, whereas a negative dval is evidence associated with a scene. The larger its magnitude, the more confident the classifier is about its prediction.

To test our hypothesis that successful recall is accompanied by reinstatement of the remembered item category, we proceeded with a two-stage approach where in the first stage a classifier was trained on the localizer data to discriminate between objects and scenes. In the second stage, it was applied to the retrieval data in order to find evidence for object or scene reinstatement. We next expand on these stages in more detail.

#### Training on localizer data (stage 1).

The classifier was trained on the broadband time series data. The single-trial voltages on nonhippocampal contacts (27 to 115 depending on participant; see *SI Appendix*, Table S1) served as features. Before applying the classifier to the retrieval data, we quantified how well object and scene images can be differentiated in the localizer. To this end, localizer data were split into training and test sets using fivefold cross-validation (75) and a separate classifier was trained for each time point in the trial. Area under the receiver operating characteristic (ROC) curve was used as the metric. The analysis was repeated five times with random folds in each iteration and results were averaged. This analysis revealed peak classification performance in the 300- to 400-ms poststimulus window (*SI Appendix*, Fig. S3). Therefore, we averaged single-trial data in the 300- to 400-ms window and trained a classifier on the localizer data that was then carried forward to stage 2.

#### Applying the classifier on retrieval data (stage 2).

To investigate the occurrence of object/scene representations in the retrieval phase (Fig. 3), the classifier was applied to each time point in the broadband retrieval trial data. This provided a time series of dvals for each trial that was subsequently subjected to statistical analysis. Since the classifier was trained on perceptual features from the localizer task, these dvals can be interpreted as a proxy for the amount of perceptual reinstatement of object and scene categories. Analyses were performed from 0 to 2 s, i.e., from recall cue onset to the end of the hippocampal gamma effect (Fig. 2).

In an additional analysis, we investigated how the timing of the switch from cue to target representation relates to hippocampal gamma power. To this end, we turned from a stimulus-locked to a gamma-peak-locked representation of the retrieval data. This was achieved by realigning each retrieval trial to its respective gamma peak. To this end, time-frequency data were normalized in each frequency band as described above and a single power time series was created by averaging z power across hippocampal contacts within the 80- to 90-Hz range. Prior to normalizing and averaging across frequencies/hippocampal contacts, a trimmed mean was used wherein power values above the 90th percentile (across trials) were discarded. We then identified local maxima in the 0- to 2.6-s trial interval. If one or more discrete gamma peaks were found in this interval, the respective time axis was realigned to the highest peak. After realigning the data this way, the same classifier trained on the localizer was applied and dvals were again subjected to statistical analysis.

### Statistics.

For behavioral analyses, RTs within participants were summarized by calculating the median in order to mitigate the effect of outliers. At the group level, arithmetic mean (M) and SEM (SEM) are reported. Paired-samples *t* tests were used to compare RTs in “Remember” and “Forgot” trials, and for object and scene cues (in “Remember” trials). Unless stated otherwise, FieldTrip’s cluster permutation test (76) was used to account for multiple comparisons for all time-frequency and classification analyses, both in sensor space and in source space (1,000 permutations). A paired-samples *t* test with a threshold of *P* < 0.05 was used to define initial clusters. Maxsum (sum of all *t* values in cluster) served as cluster statistic and Monte Carlo simulations were used to calculate the cluster *P* value (alpha = 0.05, two-tailed) under the permutation distribution. Analyses were performed at the group level.

## Supplementary Material

Supplementary File

## Data Availability

Raw data and analysis scripts are publicly available on OSF (https://osf.io/kpxw9/).
