# Supplementary material for: The hippocampus as the switchboard between perception and memory
Source: Proc Natl Acad Sci U S A. 2021 Dec 8;118(50):e2114171118. doi: 10.1073/pnas.2114171118 (PMC8685930; doi:10.1073/pnas.2114171118)
Supplement: Supplementary File [file pnas.2114171118.sapp.pdf]

Supplemental Material

Patient #	Age	Sex	Handedness	Candidate Epileptic Focus	Hemisphere	Retrieval Trials	Localiser Trials	hippocampal contacts	extrahippocampal contacts
1	24	F	R	hippocampus	L	184	100	5	27
2	35	M	R	posterior cingulate, angular gyrus, superior parietal lobe	R	192	100	2	112
3	41	M	R	hippocampus	LR	192	150	12	34
4	44	M	R	lateral temporal cortex	L	192	200	4	115
5	28	M	R	lateral temporal cortex	R	160	300	9	109
6	26	F	L	hippocampus	R	192	200	8	101
7	53	M	R	hippocampus	LR	192	200	7	41
8	29	M	R	fronto-temporal cortex	L	192	100	12	91
9	30	F	R	hippocampus	L	192	200	12	48
10	32	F	R	hippocampus	R	192	200	2	82
11	37	F	R	diffuse	~	192	200	7	93
	**34**					**188**	**177**	**7**	**78**

Table S1. iEEG patient characteristics. Bold numbers denote group averages. 'Diffuse' candidate epileptic focus indicates that invasive monitoring remained inconclusive.

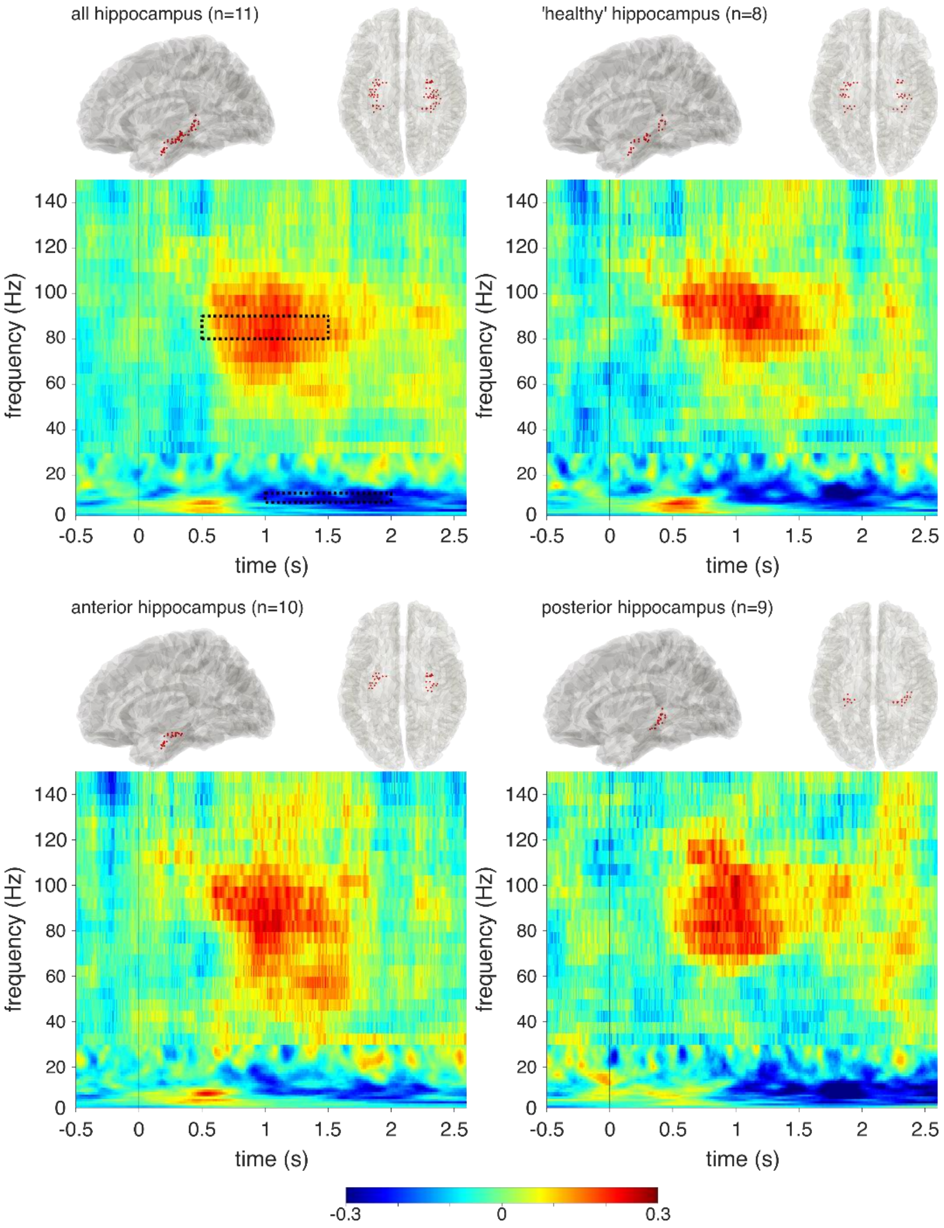

Figure S1. Time-frequency representations (TFRs) for “Remember” vs. “Forgot” trials in subselections of hippocampal contacts. *Top left*: Same as main Figure 2A and supplemental Figure S1A. Dotted rectangles demarcate time-frequency areas for “Remember” vs. “Forgot” contrasts in all subselections (gamma: 80-90 Hz, .5-1.5 s; alpha: 8-12 Hz, 1-2 s). All hippocampus: gamma: t(10) = 4.85, P = .001; alpha: t(10) = -5.28, P = .0004. *Top right*: Only hippocampal contacts located in non-candidate regions for epileptic tissue according to pre-surgical diagnostics. gamma: t(7) = 3.97, P = .005; alpha: t(7) = -3.72, P = .007. *Bottom left*: anterior hippocampal contacts (Y range in MNI space: -6 to -25). gamma: t(9) = 3.72, P = .005; alpha: t(9) = -3.98, P = .003. *Bottom right*: posterior hippocampal contacts (Y range in MNI space: -26 to -40). gamma: t(8) = 2.98, P = .018; alpha: t(8) = -4.87, P = .001.

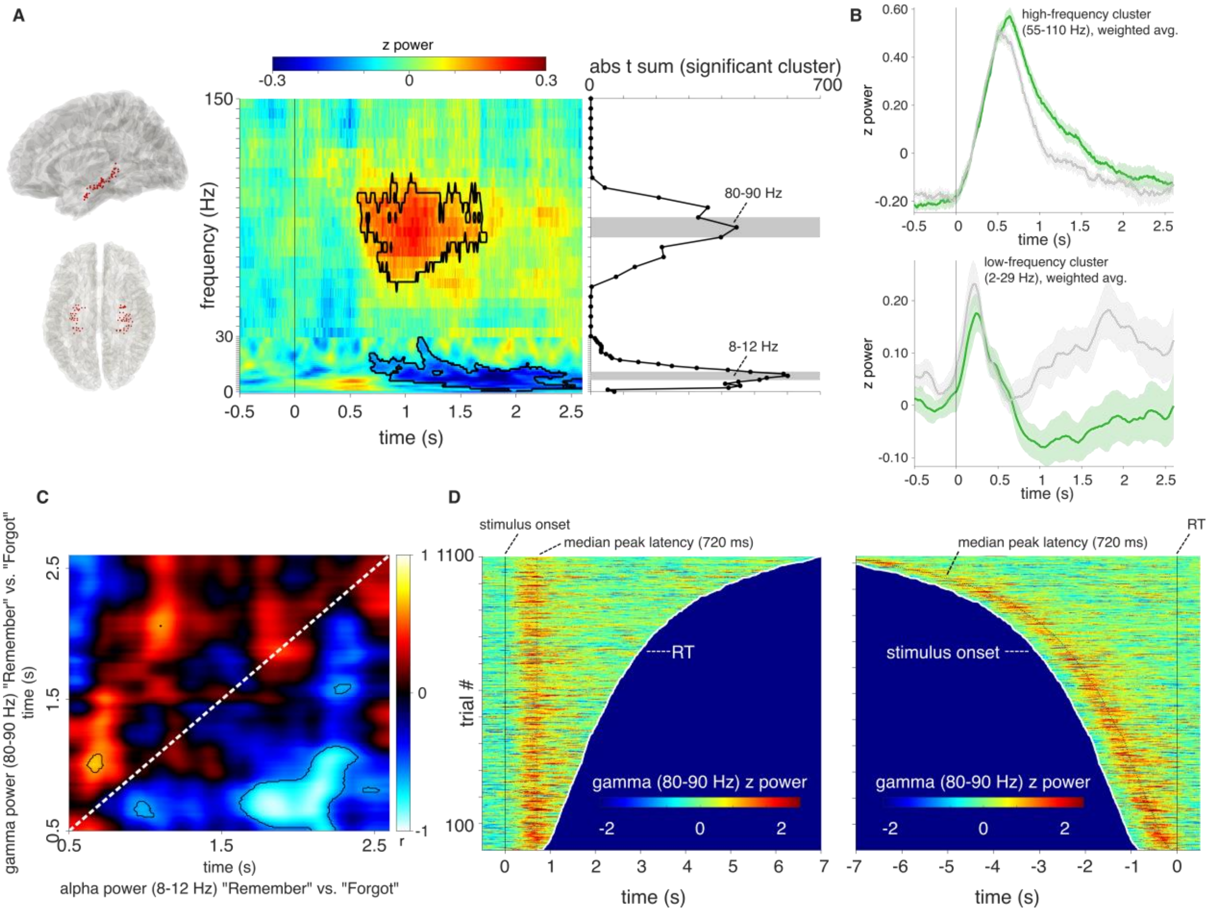

Figure S2. **A.** Unthresholded hippocampal time-frequency map. Colours depict the difference in z power for “Remember” vs. “Forgot” trials, averaged across participants. Contours highlight significant clusters (P < .05, corrected). Line plot shows absolute t values, summed across significant time points for each frequency, highlighting peaks in the alpha (8-12 Hz) and gamma (80-90 Hz) bands. **B.** Time courses for the high-frequency (*top*) and low-frequency (*bottom*) cluster, weighing the average across significant frequencies by the absolute t sum of significant time points across time. **C.** Time x time Pearson correlation across participants of gamma effects (80-90 Hz, y-axis) and alpha effects (8-12 Hz, x-axis) for “Remember” vs. “Forgot”. Contours highlight significant correlations at P < .05 (uncorrected). Time courses were smoothed with a 100 ms running average. Blue cluster below the diagonal indicates that earlier gamma increases predict later alpha decreases for “Remember” vs. “Forgot” trials. **D**. Hippocampal gamma power (80-90 Hz) across time across all “Remember” trials (pooled across participants), sorted based on trial-specific RT (white lines). *Left*: Same as main Figure 2D, from -.5 s to reaction time (RT), with dashed vertical line indicating median peak latency across all trials (720 ms). *Right*: -7 s to RT+0.5 s, with dashed vertical line indicating median peak latency across all trials.

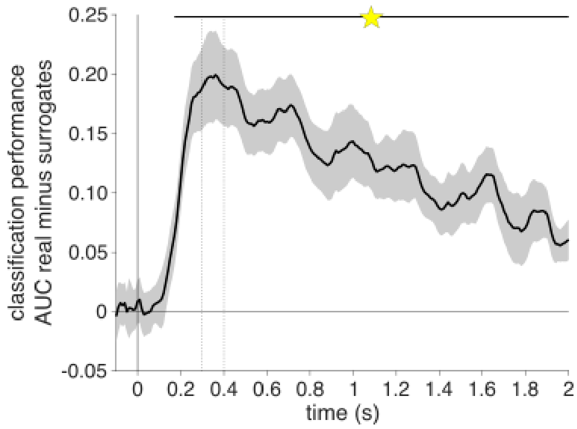

Figure S3. iEEG object vs. scene decoding. Results show mean +/-SEM of cross-validated LDA results across participants, revealing significant above-chance classification (relative to label-shuffled surrogates) from ~200 ms onwards, with peak performance from 300-400 ms (dashed vertical lines). Horizontal black line indicates statistical significance using cluster-based correction for multiple comparisons across time ($P_{cluster}$ < .001).

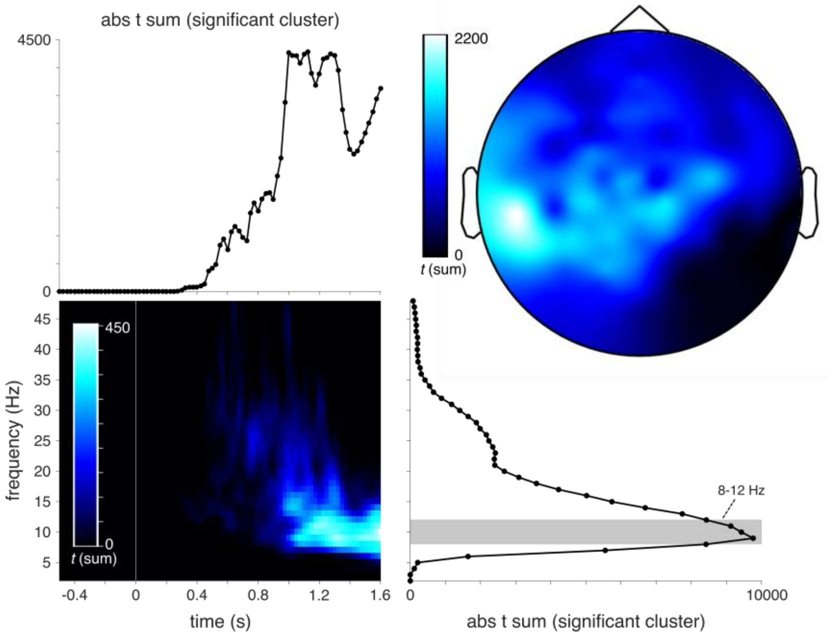

Figure S4. Extended EEG results of time x frequency x sensor comparison for “Remember” vs. “Forgot” trials, including a time range from 0-1.5 s, a frequency range from 2-48 Hz and all 128 channels. This revealed a significant time-frequency cluster (summed across significant sensors) in which alpha power (spanning frequencies in the beta range but with a peak at 9 Hz) was reduced for “Remember” vs. “Forgot” trials from ~800-1600 ms post stimulus onset ($P_{cluster}$ < .001). The scalp topography of the effect (summed across significant time/frequency bins) indicated a widespread extent, with a slightly stronger effects at left compared to right hemisphere sensors.
